# Chronic Hippocampal Abnormalities and Blunted HPA Axis in an Animal Model of Repeated Unpredictable Stress

**DOI:** 10.3389/fnbeh.2018.00150

**Published:** 2018-07-20

**Authors:** Moustafa Algamal, Joseph O. Ojo, Carlyn P. Lungmus, Phillip Muza, Constance Cammarata, Margaret J. Owens, Benoit C. Mouzon, David M. Diamond, Michael Mullan, Fiona Crawford

**Affiliations:** ^1^Roskamp Institute, Sarasota, FL, United States; ^2^Life, Health and Chemical Sciences, The Open University, Milton Keynes, United Kingdom; ^3^James A. Haley Veterans’ Hospital, Tampa, FL, United States; ^4^Departments of Psychology and Molecular Pharmacology and Physiology, University of South Florida, Tampa, FL, United States

**Keywords:** PTSD, animal model, stress, HPA axis, corticosterone

## Abstract

Incidence of post-traumatic stress disorder (PTSD) ranges from 3 to 30% in individuals exposed to traumatic events, with the highest prevalence in groups exposed to combat, torture, or rape. To date, only a few FDA approved drugs are available to treat PTSD, which only offer symptomatic relief and variable efficacy. There is, therefore, an urgent need to explore new concepts regarding the biological responses causing PTSD. Animal models are an appropriate platform for conducting such studies. Herein, we examined the chronic behavioral and neurobiological effects of repeated unpredictable stress (RUS) in a mouse model. 12 weeks-old C57BL/6J male mice were exposed to a 21-day RUS paradigm consisting of exposures to a predator odor (TMT) whilst under restraint, unstable social housing, inescapable footshocks and social isolation. Validity of the model was assessed by comprehensive examination of behavioral outcomes at an acute timepoint, 3 and 6 months post-RUS; and molecular profiling was also conducted on brain and plasma samples at the acute and 6 months timepoints. Stressed mice demonstrated recall of traumatic memories, passive stress coping behavior, acute anxiety, and weight gain deficits when compared to control mice. Immunoblotting of amygdala lysates showed a dysregulation in the p75NTR/ProBDNF, and glutamatergic signaling in stressed mice at the acute timepoint. At 6 months after RUS, stressed mice had lower plasma corticosterone, reduced hippocampal CA1 volume and reduced brain-derived neurotrophic factor levels. In addition, glucocorticoid regulatory protein FKBP5 was downregulated in the hypothalamus of stressed mice at the same timepoint, together implicating an impaired hypothalamus-pituitary-adrenal-axis. Our model demonstrates chronic behavioral and neurobiological outcomes consistent with those reported in human PTSD cases and thus presents a platform through which to understand the neurobiology of stress and explore new therapeutic interventions.

## Introduction

Upon exposure to an acute stressor, the brain initiates “fight or flight” responses involving the cardiovascular, endocrine and immune systems to prepare the body for the possible danger. After dismissal of the stressor, physiological homeostatic mechanisms restore normal baseline functions. However, a single exposure to a particularly severe traumatic stressor or prolonged exposure to milder stressors, can lead to maladaptive responses within the brain neurocircuitry regulating these homeostatic mechanisms (Bonne et al., 2004; Sherin and Nemeroff, 2011; Roth et al., 2012; Sambasivarao, 2013). Theses maladaptive responses are involved in the pathophysiology of various stress-related psychiatric illnesses such as anxiety disorders, post-traumatic stress disorder (PTSD), and depression (McEwen, 2004).

In the United States, about 13% of women and 6% of men who experience traumatic events will develop PTSD (Breslau et al., 1999). Incidence of PTSD ranges from 3 to 30% in groups exposed to specific traumatic incidents such as war, torture, or rape (Kennedy, 2007). The symptoms of PTSD are marked by the uncontrolled re-experiencing of traumatic events triggered by intrusive memories, avoidance of traumatic cues, negative alterations in cognition and mood, and dynamic fluctuation in arousal and reactivity states (DSM-5, 2013). A diagnosis of PTSD requires that these clusters of symptoms have to last for at least 1 month to demonstrate clinical impact, and they have to significantly influence the functional ability of the individual in several domains. In the past decade, a growing body of research has increased our knowledge about the pathophysiology of PTSD; including hypothalamus-pituitary-adrenal (HPA) axis dysregulation, increased sympathetic nervous system activity and cellular/molecular alterations in the hippocampus, amygdala, and prefrontal cortex. Despite these advancements, there are only a few therapeutic strategies available, and these mainly include psychotherapy and/or FDA-approved drugs designed for other psychiatric conditions such as depression and anxiety (Krystal et al., 2017). These treatments offer only symptomatic relief and usually show limited efficacy, especially in the Veteran population (Krystal et al., 2017).

To address this issue, several preclinical animal models have been developed in efforts to achieve better understanding of the underlying neurobiological abnormalities and to accelerate the drug discovery process. Modeling PTSD in animals often involves the use of intense stressors in either a single prolonged exposure, such as the single prolonged stress (SPS) paradigms (Liberzon et al., 1997), or in multiple unpredictable daily exposures such as the chronic unpredictable stress (CUS) paradigms (Deslauriers et al., 2018). While these models were successful in probing PTSD-related phenotypes, studies exploring the long-lasting behavioral effects of these paradigms in correlation with the key biological outcomes of PTSD are lacking. In addition, these models have limitations in modeling specific PTSD phenotypes such as HPA axis dysregulation. For instance, CUS and SPS paradigms often result in an increase in basal plasma glucocorticoid levels after stress cessation, while they appear to be low or unchanged in PTSD patients (Yehuda, 2001; Deslauriers et al., 2018).

We have previously shown that exposure to a single footshock with repeated exposure to TMT under restraint induced acute PTSD-like behavior including anxiety-like behavior, social avoidance and recall of fear memory in C57BL/6 mice (Ojo et al., 2014). One of the major limitations of our previous model was the lack of chronic and persistent behavioral and neurobiological changes. The presence of acute symptoms after a traumatic incident is not an accurate hallmark feature for PTSD diagnosis, as they can be attributed to a normal adaptive stress response or acute stress disorder (Flandreau and Toth, 2017). While acute stress disorder and PTSD share a number of diagnostic criteria, post-traumatic symptoms are primarily persistent (>1 month) and in PTSD may also develop after a delay (DSM-5, 2013). Therefore a chronic and long-lasting behavioral and biological outcome in a mouse model is essential to more accurately recapitulate the human PTSD experience.

To overcome this limitation in our earlier model, we intensified our previous 21-day repeated unpredictable stress (RUS) paradigm to include 10 unpredictable exposures to TMT under restraint, 5-inescapable foot shocks, and daily unstable social housing that was followed by social isolation. The presence of chronic social stress in our model is a unique feature in comparison with traditional CUS paradigms.

We show that such repeated exposures to intense physical, psychological, and psychosocial stressors resulted in PTSD-like symptomology that includes reduced weight gain, acute anxiety, and passive stress coping behavior in the forced swim test. Stressed mice were also able to recall cued fear memory at the acute timepoint, 3 and 6 months after RUS. Moreover, exposure to stress resulted in biological responses typified by neuroendocrine and HPA-axis abnormalities at a chronic time point (6 months) after RUS. Mice exposed to stress also show dysregulation in amygdaloid neurotrophic and glutamatergic signaling, emphasizing the importance of these signaling events as potential targets for modulation in treatment of PTSD.

## Materials and Methods

### Animals

C57BL/6 male mice (aged 8–10 weeks) were purchased from Jackson Laboratories (Bar Harbor, Maine) and housed in standard cages under a 12-h light/12-h dark schedule at ambient temperature. A 2-weeks habituation period was allowed before the start of any experimental procedures. All procedures were performed in accordance with OLAW guidelines under an approved protocol by the Roskamp Institute IACUC.

### The 21-Day RUS Paradigm

**FIGURE 1:**
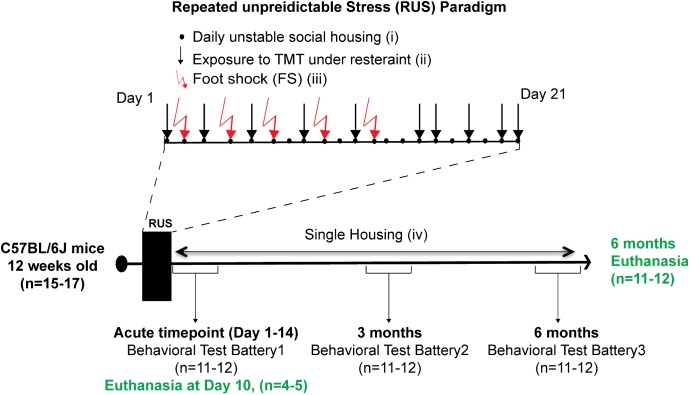
Study timeline and experimental procedures for the stress paradigm. Stress paradigm involved 21 days of daily unstable social housing with an alternate congener (i), unpredictable repetitive exposures to danger-related predator odor (fox urine, TMT), while under a decapicone restrainer for 30 min (ii), and physical trauma in the form of five repeated inescapable footshocks (iii). After 21 days of stress, animals in the stress group were singly housed until the end of the study (iv). A battery of behavioral testing was conducted at an acute timepoint (1–14 days), 3 and 6 months after the last footshock. Brain tissue and plasma were collected only at the acute timepoint (10 days) and 6 months after the last footshock.

Animals were randomly assigned to either a control group (*n* = 15) or the stress group (*n* = 17). The 21-day RUS paradigm is shown in **Figure 1**, and involved (i) daily unstable social housing with an alternate congener; (ii) unpredictable repetitive exposure to a psychological stressor of predator odor (TMT) while in a decapicone restrainer for 30 min in a dark room (1 exposure per day, 10 exposures within 21 days); (iii) physical trauma in the form of five repeated inescapable foot-shocks; and (iv) lack of social support (i.e., singly housed) post-traumatic stress. Animals in the stress group were housed individually after the 21 days, whereas animals in the control group were housed in groups of 2–3 throughout the study. Animals in the control group were never subjected to the decapicone restraint or TMT exposure, but were exposed to the fear-conditioning chamber for the same frequency and duration as the stressed mice, without administration of foot shock or auditory cues. 4–5 animals per group were euthanized at 10 days after RUS (acute timepoint) for molecular studies without receiving any behavioral testing. The remaining animals per group received a behavioral test battery at 1 day (acute timepoint) after RUS and then were retested using a similar battery at 3 and 6 months after RUS. Details about the timeline of the behavioral test battery can be found in Supplementary Figure S1. After the last behavioral experiment, all animals were euthanized and brain and plasma were collected for molecular studies (6 months timepoint).

### TMT

Trimethylthiazoline (TMT, SRQ Bio, Sarasota, FL, United States) is a component of fox urine, which we and others have previously used as a predator odor stressor (Endres and Fendt, 2009; Janitzky et al., 2015). TMT was diluted 1:10 (v/v) in double distilled water before being used as a predator odor stressor. 50 μl of 10% TMT were dispensed on a tissue paper then placed next to the restrained animal.

### Behavioral Testing

At least two baseline body weight measurements were taken during the acclimation period and before administering the stress paradigm. After exposure to the stress, body weight was measured on a weekly basis for each mouse.

### Fear Conditioning, Contextual, and Cued Fear Testing

Fear conditioning was delivered in the form of five footshocks on days 2, 4, 6, 9, and 12 of the stress exposures paradigm. This involved placing animals in a shuttle box-chamber for 3 min, with a pure tone (70 dB, 2.9 kHz) introduced during the last 30 s. At the end of the auditory cue, animals received a single 1 mA of uncontrollable/inescapable footshock for 4 s, and were allowed to recover for 1 min in the same context before returning to their home cages. The control group was allowed to explore the fear-conditioning context for 3 min without introducing a tone or electric shock. To determine retention of contextual/cued fear memory animals were tested at the acute timepoint, 3 months, and then 6 months after RUS. This involved placing animals in the fear-conditioning spatial context for 3 min and measuring the freezing response using automated video tracking system (Ethovision; Noldus—Netherlands). Freezing responses were calculated by Ethovision Software using the immobility time (2.5% immobility threshold, 20 frames per second) (Ojo et al., 2014). A cued memory test was conducted 1 h after the contextual fear memory test, and involved placing animals in a novel context for 3 min without the tone and a further 3 min with the tone. Freezing response was measured throughout the entire 6 min period.

### Open Field Test

Anxiety and sensorimotor behavior were further assessed by the open field test (Hall and Ballachey, 1932). This test was conducted in a circular maze (120 cm diameter) setup in a dim lit room (80 lux). Animals were placed in the center of the maze and the time spent in a predefined center zone and around the walls of the maze was recorded over a 15 min trial.

### Elevated Plus Maze

Anxiety behavior was evaluated using the elevated plus maze (EPM) (Pellow et al., 1985). The apparatus consists of two open and two closed arms forming a plus shape. The arms are elevated 70 cm from the floor. Each mouse was placed on the junction of the four arms of the maze, facing the open arm. The mouse was allowed to freely explore the maze for 5 min in a dark light (∼1 lux). The percentage of time spent in the open arms was calculated using Ethovision video tracking system. Animals were retested in a different room at 6 months after RUS.

### Forced Swim Test

The test was performed in a cylindrical container (20 cm height × 20 cm diameters) filled with water at room temperature. Animals were placed in the container for 6 min with video recording from the top. The time spent floating (neither swimming nor climbing) was calculated using Ethovision immobility parameter (7.5% immobility threshold, 20 frames per second) as a measure of depression-like behavior. The immobility time measured by our automated detection settings was comparable to manually calculated floating times by a blinded observer (data not shown).

### Radial Arm Water Maze (RAWM)

The RAWM test for spatial learning and memory was conducted as described by Park et al. (2008) (Soliman et al., 2010). In brief, the test was conducted in a RAWM pool (120 cm diameter and 30–40 cm height), containing six swim paths extending from an open central arena. A hidden escape platform was placed beneath the end of the target arm and each of the arms was marked with a unique visual cue. Animals were trained for 5 days with 12 trials per day to locate the hidden goal arm. Each trial lasted for 60 s. Each entry to a wrong arm was scored as a memory error. The number of errors per block trial, across the entire length of the training session was recorded using a video-tracking system (Ethovision, Noldus—Netherlands). All animals were subjected to the test at three time points after the traumatic stress (i.e., 2 weeks and 3 and 6 months after traumatic stress).

### Social Interaction and Novelty Recognition Tests (Three-Chamber Test)

The three-chamber test was used to evaluate social behavior. The testing apparatus consists of a rectangular box with three chambers separated by two doors. Each of the outer chambers contains a cage enclosure. The mice were habituated to the testing box for 5 min prior to testing. Testing occurred in two sessions. In the first session, a new intruder mouse (Stranger1) was placed in one cage, while the other cage was left empty. The tested mouse was placed in the middle chamber and left to explore the three chambers for 10 min. The time spent in the Stanger1 chamber versus the empty cage is used to evaluate social interaction of the test subject. In the second session, another intruder mouse (Stranger2) was placed in the second cage along with the previous intruder mouse (Stranger1) still held in the first cage, and the test subject was allowed to freely explore both chambers for 10 min. The time spent in the Stanger2 versus Stranger1 chamber was used to evaluate the social memory of the tested animal.

### Enzyme-Linked Immunosorbent Assay (ELISA)

To obtain blood specimens, animals were anesthetized with isoflurane, and approximately 500 μL of blood were collected into EDTA tubes by cardiac puncture immediately prior to euthanasia. Samples were centrifuged at 3000 ×*g* for 3 min, and plasma samples (clear supernatant fraction) were flash frozen in liquid nitrogen and stored at –80°C. Plasma Corticotropin-releasing hormone (CRH) levels were measured using an ELISA purchased from Life-Span Biosciences (Seattle, WA, United States). The Adrenocorticotropic Hormone (ACTH) ELISA was purchased from Cloud-Clone Corp. (Katy, TX, United States). Tissue and plasma corticosterone levels were measured using an ELISA from Arbor Assays (Ann Arbor, MI, United States) and tissue brain-derived neurotrophic factor (BDNF) levels were measured using an ELISA from Boster Bio (Pleasanton, CA, United States). ELISA kits were used as per manufacturers’ instructions.

### Brain Tissue Preparation and Western Blotting

Brains from six animals per group were used for biochemical analysis while the brains form the remaining animals were used for hippocampal volume estimation (histopathology). Western blot experiments were conducted as described previously (Ojo et al., 2016). In brief, six animals from the stress group and six control animals were used for all western blot and ELISA experiments from brain tissue. Tissue was collected following transcardial perfusion by gravity drip with phosphate buffered saline (PBS). Both hemispheres were extracted and dissected into multiple regions (hippocampus, amygdala, and hypothalamus); these were flash frozen in liquid nitrogen and kept at –80°C.

For western blotting analyses, the hippocampi or amygdala from both hemispheres were homogenized in 200 μL of M-PER protein extraction reagent containing proteinase and phosphatase inhibitors (Thermo fisher) using a probe sonicator. Samples received 2-s long sonication pulse followed by incubation on ice for 10 s, this process was repeated three times. Homogenized samples were spun in a centrifuge at 15,000 ×*g* for 10 min and tissue supernatants were collected. Supernatant fractions were denatured at 99°C by boiling in Laemmli buffer (Bio-Rad) containing DDT. Samples were then subsequently resolved on 4–15% gradient polyacrylamide criterion gels (Bio-Rad). After electrotransferring, polyvinylidene difluoride membranes were blocked in 5% milk made in Tris-buffered saline and subsequently immunoprobed for different brain-specific primary antibodies overnight (Supplementary Table S1). After three washing steps, membranes were probed with horseradish peroxidase-linked secondary antibodies (Supplementary Table S1). Anti-GAPDH antibody was used as a housekeeping gene to quantify the amount of proteins electrotransferred, and signal intensity ratios were quantified by chemiluminescence imaging with the ChemiDocTM XRS (Bio-Rad).

### Multiplex Enzyme-Linked Immunosorbent Assay (ELISA)

Levels of nine cytokines; interferon γ (IFN-γ), interleukin (IL)-1β, IL-2, IL-4, IL-6, IL-10, IL-12p70, IL-13, and tumor necrosis factor α were measured from a single plasma sample (12.5 μL) using the MSD proinflammatory Panel I multiplex assay (MesoScale Discovery, Gaithersburg, MD, United States).

### Neuropeptide Magnetic Bead Panel From Hypothalamus

Levels of six neuropeptides (α-MSH, β-Endorphin, Neurotensin, Orexin A, Oxytocin, Substance P) were measured in the hypothalamus using MILLIPLEX MAP Rat/Mouse Neuropeptide Magnetic Bead Panel. The following procedures were implemented in order to extract neuropeptides from the hypothalamic tissue. Each dissected hypothalamus was dissolved in 200 μL deionized water and homogenized using a probe sonicator on ice. Immediately after homogenization, 100 μL of the hypothalamus lysates were mixed with 200 μL acetonitrile and incubated for 10 min at room temperature then centrifuged at 17,000 ×*g* for 5 min. Clear supernatants were collected and dried using speed-vac without sample heating then solubilized in 100 μL of the manufacture’s assay buffer. The rest of multiplex assay steps were carried out as instructed by the manufacturer’s guide. Phosphatase and protease inhibitors were added to the remainder of the original lysates and stored for western blot experiments.

### Hippocampal Volume Estimation

Five control animals and six stressed animals where used for volume estimation from the hippocampus using Cavalieri principal. At 6 months after last stress exposure, mice were anesthetized with 3% isoflurane and transcardially perfused with a 4% paraformaldehyde PBS solution. Brain hemispheres were post-fixed overnight in 4% paraformaldehyde-PBS solution. The volume of the right hippocampus as guided by bregma coordinates (–0.94 to –3.28 mm) was determined by quantitative light microscopy using the Cavalieri method as described elsewhere (Ojo et al., 2014). In brief, sagittal sections from the extent of the right hippocampus of each animal (taking every 20th serial section) were mounted onto superfrost plus slides. An average of 10–15 sections were collected per animal. Mounted sections were air-dried and stained with a solution of 0.1% cresyl fast violet (Tedpella, Reading, CA, United States) and were viewed at low magnification using a DP72 digital camera attached to a motorized Olympus BX63 digital photomicroscope (Olympus, Center Valley, PA, United States). Analyses were carried out blind and were made via the Cell SENS Olympus software package. Digital images were captured electronically and the boundaries of the total dorsal hippocampal compartment and its subfields (DG, CA1, and CA2/CA3) were digitally outlined on each section from the series of sagittal sections of the right hemisphere. For each animal, the total volume of the right hippocampus and its subfields were subsequently derived by multiplying the calculated total surface area by the mean distance between the series of sections. Data are expressed as mean volume (in mm^3^) ± SEM.

### Statistical Analysis

The relationships between stress and control animals for Western blotting, ELISA, and neurobehavioral data were assessed using a *t*-test predefined criterion of *p* < 0.05 to assess group differences, while experiments examining stress effects over time were assessed using a RM-ANOVA. Pearson correlation and Spearman correlation were used for parametric and non-parametric data sets, respectively. The choice of the appropriate statistical test was made after assessment of normal distribution using the Shapiro–Wilk normality test. All analyses were performed with Graph Pad prism version 7.0a statistical software (La Jolla, CA, United States). Statistical outliers were identified using Grubbs’ test and excluded.

## Results

### Mice Exposed to the 21-Day Stress Paradigm Show Attenuation of Bodyweight Gain and Anxiety-Like Behavior Immediately After Stress

**FIGURE 2:**
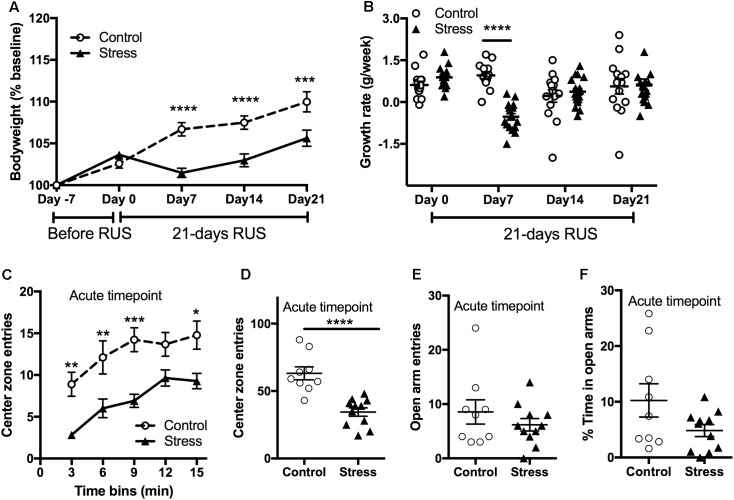
Effect of RUS on bodyweight gain and anxiety-like behavior. During the 21-day stress paradigm, stressed mice showed a reduction in body weight gain when compared to control mice **(A)**. Growth rate was significantly lower only during and after the first week of stress (day 7) **(B)**. Exposure to stress increased anxiety-like behavior at the acute timepoint as evident by reduced center zone entries per a 3-min time bin **(C)** and in the total 15 min **(D)** in the open field test. No significant changes were observed in the EPM open arm time **(E)** and entries **(F)** at the acute timepoint. Data in **(A,B)** was analyzed using repeated measures Two-Way ANOVA followed by *post hoc* Tukey’s test (*n* = 14–17), while data in **(C–F)** were analyzed using a student *t*-test (*n* = 8–9). Asterisks denote statistical significance as follows: ^∗^*p* < 0.05; ^∗∗^*p* < 0.01; ^∗∗∗^*p* < 0.001; ^∗∗∗∗^*p* < 0.0001.

Stress is usually associated with changes in bodyweight and food intake (Harris, 2014). Therefore, we obtained the weekly bodyweight for each mouse to examine the effects of the RUS paradigm (**Figure 2A**). Stressed mice showed reduced weight gain in the first 3 weeks of stress (RM-ANOVA: interaction *F*_(4,116)_ = 12.54, *p* < 0.0001, with the Sidak *post hoc* test) compared to their unexposed group. The changes in bodyweight are mainly driven by an intense decrease in their growth rate during the first week of stress (RM-ANOVA: interaction *F*_(3,87)_ = 10.58, *p* < 0.0001, with the Sidak *post hoc* test, **Figure 2B**). The bodyweight deficits in the stressed mice persisted until 2 months after stress (*t*-test: *t*_(20)_ = 2.54, *p* = 0.02) but by 3 and 6 months post-stress there are no significant differences in bodyweight between groups (Supplementary Figure S2). In addition, stressed mice showed an anxiety-like behavior as evident by reduced center zone entries per 3 min time bins (RM-ANOVA: stress effect *F*_(1,18)_ = 27.87, *p* < 0.0001, with the Sidak *post hoc* test) and throughout the entire 15 min (*t*-test: *t*_(18)_ = 5.174, *p* < 0.0001) in the open field test (**Figures 2C,D**) at the acute timepoint. However, assessment in the EPM in the same timepoint showed no significant differences between groups in the open arm duration (*t*-test: *t*_(18)_ = 1.8, *p* = 0.083, **Figure 2E**) or open arm entries (*t*-test: *t*_(18)_ = 1.8, *p* = 0.99, **Figure 2F**).

### Stressed Mice Recall Fear Memory and Show Passive Stress Coping Behavior at 6 Months After RUS

**FIGURE 3:**
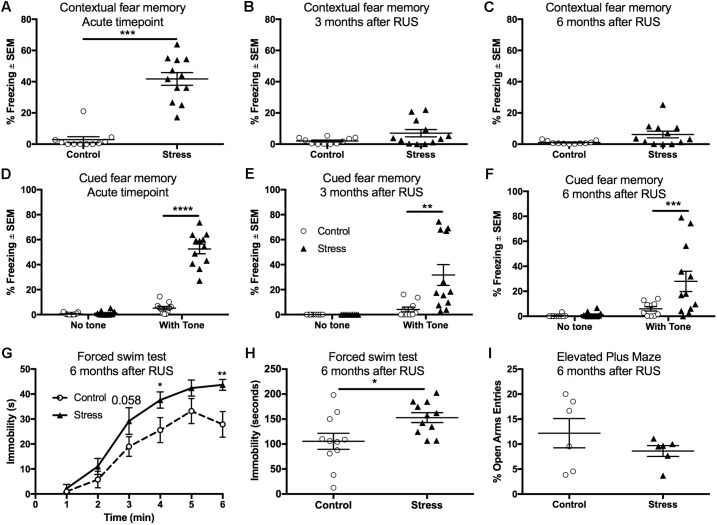
Recall of contextual and cued fear memory at the acute timepoint, 3 and 6 months after RUS. Stressed animals recalled contextual fear memories only at the acute timepoint as evident by increased freezing time compared to controls at the acute timepoint **(A)**, but not at 3 months **(B)** or 6 months **(C)** after RUS. Panels **(E–G)** depict% freezing scores in the first minute of the cued memory test in absence and presence of a cue (tone) at the acute, 3- and 6-month timepoints, respectively. There was no significant freezing for all treatment groups when tested in a new context without cues (no tone) at all timepoints **(D–F)**. After tone introduction, RUS mice showed a significant increase in % freezing at the acute timepoint, 3 and 6 months after RUS **(D–F)**. Stressed mice displayed increased immobility time in minutes 4 and 6 of the FST when compared to a control group **(G)**. Average immobility time in the last 4 min was increased in stressed mice compared to the control group **(H)**. RUS didn’t alter open arm entries in the elevated plus maze test at 6 months after stress. Data in **(A–C,H,I)** were analyzed using a student *t*-test (*n* = 6–12), while data in **(D–F)** were analyzed using repeated measures Two-Way ANOVA followed by *post hoc* Sidak test (*n* = 10–12). Asterisks denote statistical significance as follows: ^∗^*p* < 0.05; ^∗∗^*p* < 0.01; ^∗∗∗^*p* < 0.001; ^∗∗∗∗^*p* < 0.0001.

Fear conditioning using electric footshocks was used as a stressor in the RUS paradigm and also as a tool to evaluate the recall of contextual and cued fear memory responses (Ojo et al., 2014). Stressed mice showed a significant increase in their freezing (immobility time) when placed in the same trauma context at the acute timepoint (*t*-test: *t*_(21)_ = 8.4, *p* < 0.0001, **Figure 3A**). However, stressed mice were not able to recall contextual fear memory when examined at 3 and 6 months after RUS (**Figures 3B,C**, *p* > 0.05). We also examined recall of cued fear memory at the same timepoints. Mice were placed in a novel context for 3 min, and then exposed to a trauma related cue (auditory tone) lasting an additional 3 min. While all animal groups showed minimal freezing during the first 3 min in the novel context (without a tone), stressed animals showed an increased % freezing upon tone introduction at the acute timepoint (Two-way ANOVA, interaction *F*_(1,40)_ = 111.8, *p* < 0.0001, **Figure 3D**), 3 months (Two-way ANOVA, interaction *F*_(1,40)_ = 8.7, *p* < 0.0052, **Figure 3E**) and 6 months (Two-way ANOVA, interaction *F*_(1,40)_ = 5.4, *p* < 0.025, **Figure 3F**) after RUS. Chronic changes in stress-coping strategies and anxiety-like behavior were also examined at 6 months after RUS using the forced swim test and the EPM, respectively. As depicted in **Figures 3G,H**, stressed animals showed negative stress coping behavior as evident by increased immobility time in the forced swim test (*t*-test: *t*_(20)_ = 2.5, *p* = 0.02, **Figure 3H**). No significant differences were observed between control and stressed mice at 3 and 6 months after RUS in the number of open arm entries in the EPM (*p* > 0.05; Supplementary Figure S3A and **Figure 3I**) or center zone entries in the open field test (*p* > 0.05; Supplementary Figure S3B). It is important to note that the performance of mice in the EPM test and the suitability of the EPM and Open field for retesting is questionable (Walf and Frye, 2007; Hussin et al., 2012). Control mice in the EPM always performed poorly (spending only few seconds in the open arms) which reduced the window for detecting a significant effect between sham and stressed mice. Additionally, although some studies suggested changing the retesting room and separating the trials by 4 weeks to improve retesting reliability (Schneider et al., 2011), our data indicated that control mice show fewer entries to the open arms of EPM and center zone of the Open Field upon retesting at different rooms 3 and 6 months after RUS (Supplementary Figures S3A,B).

Contextual memory retrieval is thought to be a hippocampal-dependent task (Maren and Holt, 2000). Since stressed mice were able recall cued fear memory, but not contextual fear memory at 3 and 6 months after RUS, we examined the effect of stress on other hippocampal-related tasks such as social memory and spatial learning. Mice exposed to RUS did not show any impairment in social memory at the acute timepoint, 3 months or at 6 months after RUS when tested using social novelty recognition tests (*p* > 0.05; Supplementary Figure S4). Our findings in the social memory test at the chronic timepoints are inconclusive because control mice did not show any preference to the novel mouse (Stranger2), possibly due to retesting (Supplementary Figure S4B). There was, however, no impairments in spatial learning and memory in stressed animals at any timepoint when tested using the RAWM (*p* > 0.05; Supplementary Figure S5).

### Stressed Mice Display Lower Plasma Corticosterone Levels 6 Months After RUS; Suggesting Blunted HPA-Axis Reactivity

**FIGURE 4:**
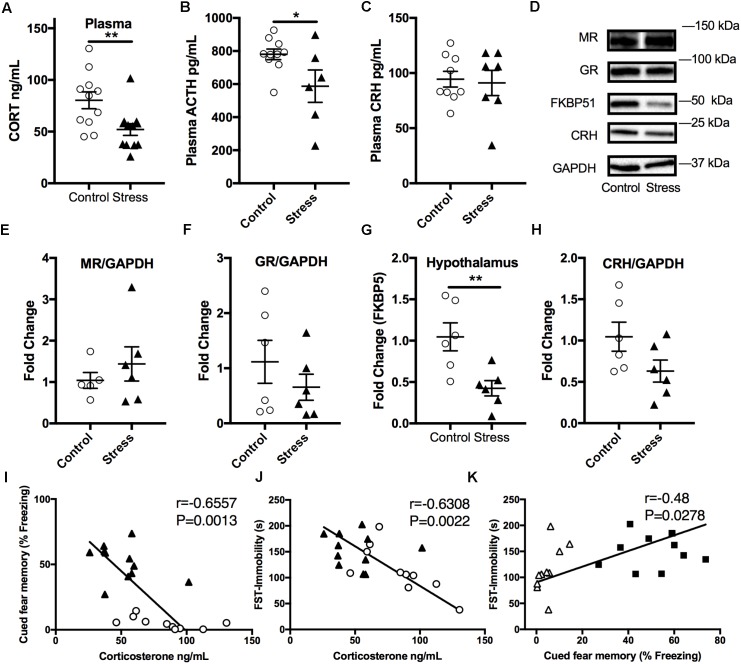
Effect of stress on HPA axis regulation at 6 months after RUS. Mice exposed to stress showed lower plasma levels of corticosterone **(A)** and ACTH **(B)**, but not CRH **(C)** when compared to a control group at 6 months after RUS (*n* = 8–12). Representative western blot images for markers of glucocorticoid signaling in the hypothalamus **(D)**. Quantification of mineralocorticoid Receptor **(E)**, glucocorticoid receptor **(F)**, FKBP5 **(G),** and CRH **(H)** levels in the hypothalamus (*n* = 5–6). Only the levels of FKBP5 **(G)** were significantly reduced after RUS. Plasma corticosterone levels were inversely correlated with the% freezing scores in the cued fear memory test **(I)** and the immobility time in the forced swim test **(J)** (*n* = 21). There was a positive correlation between% freezing scores in the cued fear memory test and the immobility time in the forced swim test **(K)**. Each solid black triangle represents an animal in the stress group, while white circles represent control animals. Asterisks denote statistical significance as follows: ^∗^*p* < 0.05; ^∗∗^*p* < 0.01.

In order to examine the effect of stress on the HPA axis, baseline plasma corticosterone levels were measured at the end of the light cycle 6 months after RUS. We found that mice exposed to stress showed significantly lower plasma corticosterone levels (*t*-test: *t*_(21)_ = 2.9, *p* = 0.009, **Figure 4A**) compared to control mice. Similarly, ACTH levels were significantly lower in stressed mice compared to controls (*t*-test: *t*_(14)_ = 2.3, *p* = 0.04, **Figure 4B**). No significant change was seen in plasma CRH at 6 months (*p* > 0.05, **Figure 4C**). We subsequently examined glucocorticoid signaling in the hypothalamus by measuring the expression levels of glucocorticoid receptors (GRs) and regulatory proteins (**Figures 4D–H**). While there was no change in expression levels of GR, mineralocorticoid receptor (MR), or CRH levels, the levels of FK506 binding protein 51 (FKBP51) were downregulated in the hypothalamus of stressed mice relative to controls (*t*-test: *t*_(9)_ = 2.4, *p* = 0.042, **Figures 4D,G**). To explore the relationship between abnormalities in the HPA axis function and behavioral outcomes in our model, we assessed the statistical correlation between individual mouse corticosterone plasma levels and their corresponding neurobehavioral scores in both control and stress groups. We found a significant negative correlation between plasma corticosterone levels and freezing scores in the cued fear memory test (Spearman correlation: *r* = –0.749, *p* = 0.001, **Figure 4I**) and the immobility time in the forced swim test (Pearson correlation: *r* = –0.63, *p* = 0.0022, **Figure 4J**). Furthermore, cued fear memory freezing scores correlated positively with the immobility time in the forced swim test (Pearson correlation: *r* = –0.479, *p* = 0.0278, **Figure 4K**).

Previous studies have suggested interplay between the endocrine and inflammatory/immune systems in the pathophysiology of depression (Griffin et al., 2014) and PTSD (Griffin et al., 2014), owing partly to the immunoregulatory role of corticosterone. To examine the effect of our RUS paradigm on the peripheral immune system, we evaluated the levels of plasma cytokines at 6 months after stress. We found a twofold increase in plasma levels of pro-inflammatory IL-1β (*t*-test: *t*_(8)_ = 2.4, *p* = 0.04, **Table 1**) and IFN-γ (*t*-test: *t*_(7)_ = 3.9, *p* = 0.006, **Table 1**), while the levels of other cytokines were not significantly altered (**Table 1**).

**Table 1:** Effect of stress on plasma cytokines at 6 months after RUS.

Cytokine	Fold change^$^ ± SD	*p*-Value	*n*	Time after stress (months)
IFN-γ^∗^	1.97 ± 0.26	0.04^∗^	5	6
TNFα	1.02 ± 0.82	0.88	5	6
IL-1β^∗∗^	1.94 ± 0.07	0.006^∗∗^	5	6
IL-2	1.15 ± 0.27	0.688	5	6
IL-5	0.84 ± 0.57	0.613	5	6
IL-6	1.25 ± 1.37	0.431	5	6
IL-10	0.83 ± 1.30	0.132	5	6
IL-12	1.65 ± 4.96	0.276	5	6


*^$^Plasma cytokines levels in stressed animals relative to controls ± SD. Asterisks denote statistical significance as follows: ^∗^*p* < 0:05; ^∗∗^*p* < 0:01.*

**FIGURE 5:**
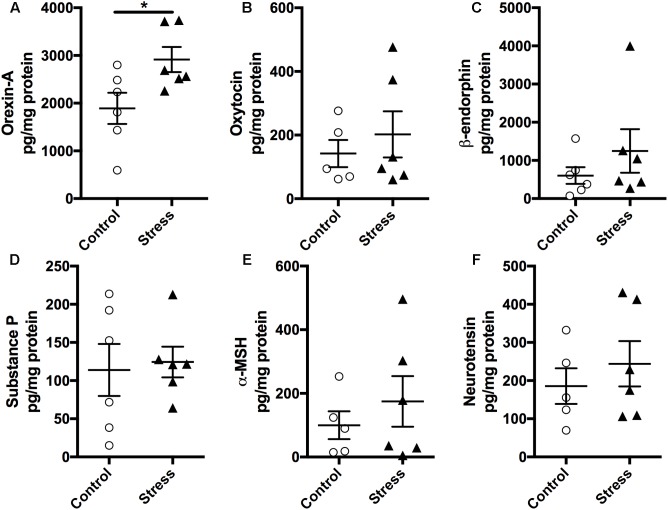
Exposure to stress elevates **(A)** Orexin-A levels in the hypothalamus. Magnetic beads multiplex assay showed an increase in the hypothalamic levels of Orexin-A at 6 months after RUS. No significant changes were observed in the levels of Oxytocin **(B)**, β-endorphin **(C)**, Substance P **(D)**, α-MSH **(E)**, and Neurotensin **(F)**. Data in **Figure 5** were analyzed using a student *t*-test (*n* = 5–6). Asterisks denote statistical significance as follows: ^∗^*p* < 0.05.

As the hypothalamus secretes numerous neuropeptides that regulate several autonomic functions implicated in regulating neurocircuitry pathways involved in stress management, we measured the expression levels of various neuropeptides in the hypothalamus using a magnetic bead multiplex assay. As shown in **Figure 5**, there was no significant difference in the expression levels of Oxytocin, β-endorphin, Neurotensin, α-MSH, and Substance P (**Figure 5**, *p* > 0.05). Interestingly, Orexin-A levels were significantly elevated in stressed mice compared to controls (*t*-test: *t*_(10)_ = 2.437, *p* = 0.035, **Figure 5A**).

### Effect of Stress on the Hippocampus at 6 Months After RUS

**FIGURE 6:**
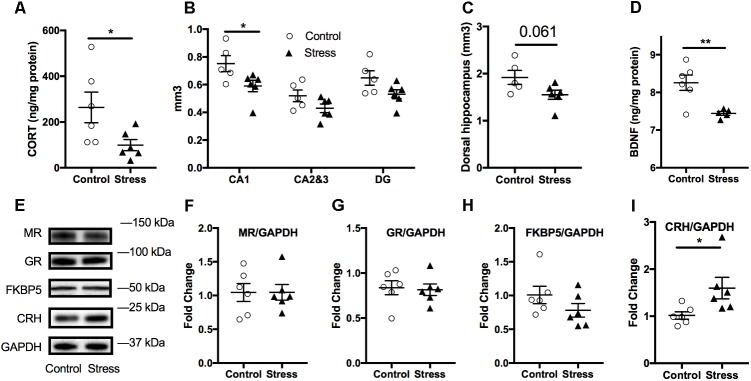
Effect of stress on the hippocampus at 6 months after RUS. Effect of stress on the volume of the dorsal hippocampus **(A)** and the volume other hippocampal regions **(B)**. Stress resulted in a volume reduction only in the CA1 region **(B)**. Hippocampal BDNF **(C)** and corticosterone **(D)** levels were also reduced at 6 months after stress. Representative western blot images for markers of glucocorticoid signaling in the Hippocampus **(E)**. Quantification of mineralocorticoid receptor **(F)**, glucocorticoid Receptor **(G)**, FKBP5 **(H)**, and CRH **(I)** levels in the hippocampus at 6 months after RUS. Only the levels of CRH **(I)** were significantly reduced after RUS. Data were analyzed using a student *t*-test (*n* = 5–6). Asterisks denote statistical significance as follows: ^∗^*p* < 0.05; ^∗∗^*p* < 0.01.

The hippocampus is prone to negative consequences of stress (McEwen et al., 2016) and it is postulated that corticosteroids are primary mediators of these stress effects (Roth et al., 2012). Consistent with our plasma findings, corticosterone levels were also reduced in the hippocampal tissue lysates of stressed mice compared to controls (*t*-test: *t*_(10)_ = 2.34, *p* = 0.044, **Figure 6A**). To further examine the chronic effect of RUS on the hippocampus, we estimated the volume of the hippocampus and its subfields and measured the levels of a number of synaptic plasticity markers. Using the Cavalieri stereology principle for volume estimation, we found that the volume of the CA1 region of the dorsal hippocampus (*t*-test: *t*_(9)_ = 2.34, *p* = 0.044, **Figure 6B**) was significantly reduced in stressed animals compared to controls at 6 months after stress. In addition, there was a trend toward reduced total dorsal hippocampus volume (*t*-test: *t*_(9)_ = 2.13, *p* = 0.061, **Figure 6C**) in stressed animals, and no significant change in CA2 and CA3 or Dentate gyrus regions (*p* > 0.05, **Figure 6B**). We then examined the expression levels of notable markers/mediators of neuroplasticity using immunoblot analysis of hippocampal lysates. BDNF levels were downregulated in mice exposed to RUS (*t*-test: *t*_(9)_ = 3.55, *p* = 0.006, **Figure 6D**), while the levels of other markers such as Post Synaptic Density protein 95 (PSD95), CAMKII, NGF, TRKB, ProBDNF, and NMDA receptors were not significantly altered after RUS (*p* > 0.05; Supplementary Figure S6). Interestingly, CRH levels were elevated in animals exposed to stress (*t*-test: *t*_(10)_ = 2.4, *p* = 0.037, **Figure 6I**) at 6 months. Moreover, we did not observe any significant change in glucocorticoid signaling receptors (GR and MR) or related binding proteins (FKBP51) at 6 months (**Figures 6E–I**).

### Effect of Stress on Markers of Neuroplasticity in the Amygdala

**FIGURE 7:**
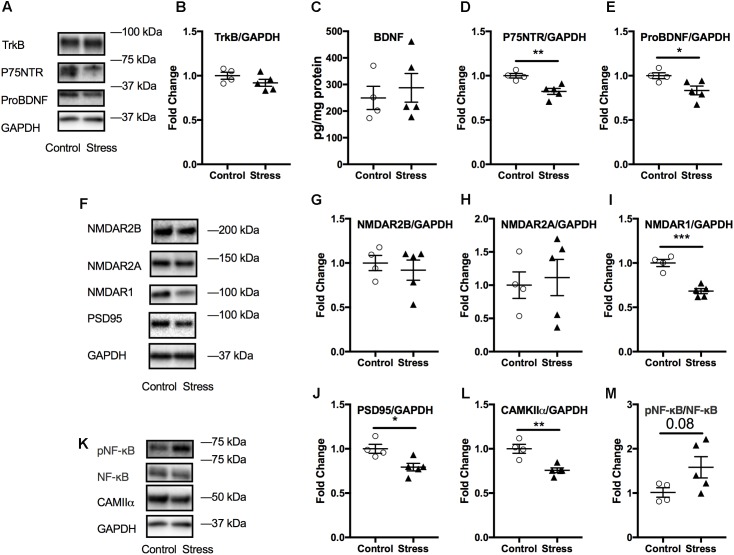
Effect of stress on the amygdala at the acute timepoint. Representative western blot images from amygdala lysates for neurotrophic markers, NMDA receptors, and other synaptic plasticity markers are shown in **(A,F,K)**, respectively. Quantification of western blot images of TRKB **(B)**, P75NTR **(D)**, ProBDNF **(E)**, and NMDAR2B **(G)**, NMDAR2A **(H)**, NMDAR1 **(I)**, PSD95 **(J)**, CAMKII **(L)**, and pNF-κB/NF-κB **(M)** levels in the amygdala at the acute timepoint. No change in amygdala BDNF levels after RUS at the acute timepoint **(C)**. All Data were analyzed using a student *t*-test (*n* = 5–6). Asterisks denote statistical significance as follows: ^∗^*p* < 0.05; ^∗∗^*p* < 0.01; ^∗∗∗^*p* < 0.001.

The amygdala is associated with emotional learning and cued fear memory and is also prone to negative consequences of stress (McEwen et al., 2016). Therefore, we probed for the levels of a number of markers associated with neuroplasticity using immunoblotting. No significant changes were observed in amygdala lysates at 6 months after stress (*p* > 0.05; Supplementary Figure S7). However, we observed that lysates from the acute timepoint showed significant changes in several markers associated with neuroplasticity. As shown in **Figure 7**, there was a significant reduction in the levels of ProBDNF (*t*-test: *t*_(7)_ = 2.64, *p* = 0.034, **Figure 7E**) and its signaling receptor, P75NTR (*t*-test: *t*_(7)_ = 4.02, *p* = 0.0052, **Figure 7D**). In addition, levels of NMDA receptor type1 were reduced (*t*-test: *t*_(7)_ = 6.15, *p* = 0.0003, **Figure 7I**), including downstream signaling kinase, CAMKII (*t*-test: *t*_(7)_ = 4.5, *p* = 0.003, **Figure 7L**) and spine density protein marker, PSD95 (*t*-test: *t*_(7)_ = 3.17, *p* = 0.016, **Figure 7J**) in animals exposed to stress compared to controls.

**FIGURE 8:**
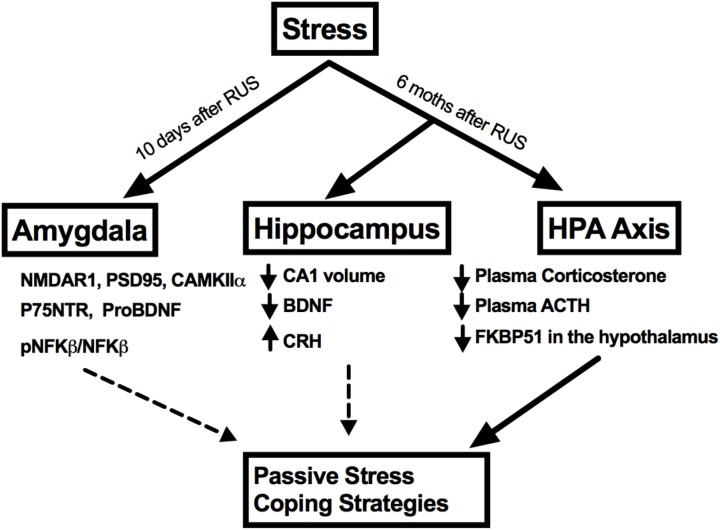
A summary of stress effects on the amygdala, hippocampus, and HPA axis at different timepoints after RUS.

No significant changes were observed in the amygdalar levels of BDNF or its receptor, Trkb (*p* > 0.05, **Figures 7B,C**); neither were there any changes in other NMDA receptor subunits such NMDAR2B and NMDAR2A (*p* > 0.05, **Figures 6G,H**) or in the ratio of phosphorylated nuclear factor κB to nuclear factor κB (pNF-κB/NF-κB). A summary of the findings presented in this study is depicted in **Figure 8**.

## Discussion

In this study, we assessed the chronic neurobehavioral, neuroendocrine, and neurobiological consequences of a RUS paradigm in mice. The purpose of our study was to develop a mouse model of stress with relevance to PTSD that demonstrates chronic behavioral deficits (face validity) with validated pathophysiological measures (construct validity). Using a battery of behavioral tests, we showed that animals exposed to RUS demonstrate traits similar to the human condition; particularly acute anxiety (**Figures 2C,D**), recall of traumatic memory cues (**Figures 3D–F**), and negative alterations in stress coping strategies (**Figures 3G,H**) at 6 months after RUS.

Blunted HPA axis responsiveness (Yehuda et al., 1993; Yehuda, 2001) (measured as lower cortisol levels compared to control subjects) and reduced hippocampal volume (Bremner et al., 1995; Levy-Gigi et al., 2013) have been frequently reported in PTSD human subjects. Our findings of lower plasma corticosterone levels and reduced hippocampal CA1 volume 6 months after RUS (**Figures 4A, 6B**) support the construct validity of our model.

Because we have utilized a prolonged unpredictable repeated stress paradigm in this study, a caveat of this work is the ability to distinguish between PTSD and other related neuropsychiatric conditions such as depression. Although there is a considerable symptom overlap between PTSD and depression, the nature of HPA axis dysregulation is markedly different (Schöner et al., 2017). Increased plasma cortisol levels is a common finding in human patients with depression (Palazidou, 2012; Gold et al., 2015). In animal studies, exposure to chronic mild to moderate stress is commonly used to study depression (McEwen, 2004; Strekalova et al., 2004). For example, CUS paradigms involve random, unpredictable exposure to variable daily stressors such as restraint, cage tilting and shaking for 4–8 weeks. CUS models typically show anxiety, depression-like behavior and increased plasma corticosterone levels at one, 6 and 18 days after stress cessation (Monteiro et al., 2015; Abd El Wahab et al., 2018). Interestingly, C57BL/6 mice appear to be more resilient to CUS compared to other mouse strains and longer stress durations are usually needed to show depression-like behavior (Monteiro et al., 2015). Blunted HPA axis reactivity to acute stressors has been reported in repeated immobilization (Liberzon et al., 1997; Harvey et al., 2006), social defeat (Beitia et al., 2005), and predator exposure models (Zoladz et al., 2015). In addition, social isolation has been linked to reduced plasma baseline corticosterone levels in mice (Arndt et al., 2009; Martin and Brown, 2010), therefore the incorporation of repeated stress and social isolation to our RUS paradigm may have contributed to the decreased basal corticosterone levels in our model as compared to the traditional CUS paradigms. Future studies including earlier timepoints and control groups receiving CUS and SPS are needed to explore the differences between these paradigms and RUS. In contrast to depression, PTSD patients typically show lower plasma cortisol levels compared to healthy controls (Yehuda et al., 1993; Yehuda, 2001). While one possible interpretation of our behavioral results is stress induced depression, the blunted HPA axis reactivity is consistent with observations in PTSD, and thus the lower plasma corticosterone levels observed at 6 months after RUS suggest that our model has more relevance for PTSD.

The blunted HPA axis response in PTSD is thought to be due to increased negative feedback sensitivity via enhanced GR responsiveness (Yehuda et al., 2009; Hartmann et al., 2012; Schöner et al., 2017). FK506 binding protein 5 (FKBP5) is thought to be a regulator of GR sensitivity. Under normal conditions, cortisol binds GR in the cytoplasm and results in GR dissociation from chaperone proteins such as heat shock 90 and FKBP5 inside the cytosol, resulting in the transport of GR into the nucleus through openings in the nuclear pores, the subsequent activation of GR transcriptional activity and increase of regulatory proteins to promote negative feedback mechanisms. In contrast, with PTSD, less free cortisol is available to bind GR, which in turn results in downregulation of negative feedback regulatory proteins such as FKBP5 (Girgenti et al., 2017b). Interestingly, FKBP5 gene knockout mice show increased GR sensitivity resulting in decreased corticosterone levels after restraint stress (Touma et al., 2011). Previous reports have shown that FKBP5 expression levels are reduced in white blood cells and brains of trauma-exposed patients (Yehuda et al., 2009; Levy-Gigi et al., 2013; Young et al., 2015). FKBP5 levels were also reduced in the prefrontal cortex of post-mortem PTSD human subjects (Girgenti et al., 2017a). In addition, FKBP5 gene polymorphism and epigenetic methylation patterns are associated with GR sensitivity and have also been linked to early-childhood trauma and risk of PTSD (Yehuda et al., 2009; Zovkic and Sweatt, 2012). We found the levels of FKBP5 to be reduced in the hypothalamus of RUS mice the 6 months timepoint, supporting a central role for FKBP5 in HPA axis dysregulation in our model (**Figure 4D**). These findings together with the reductions in plasma corticosterone and ACTH levels (**Figures 4A,B**) at 6 months after RUS, implicates a dysfunction in the intrinsic homeostatic mechanisms regulating the HPA axis and also highlights the presence of possible molecular abnormalities in the hypothalamus as a contributing factor underlying this dysfunction. Previous reports have also suggested a role for the pituitary gland in dysregulation of HPA axis sensitivity in PTSD patients (Ströhle et al., 2008; Cooper et al., 2017), however, a full understanding of the nature of this dysregulation remains elusive. Further chronic studies involving challenging the hypothalamus, pituitary and/or adrenal glands after exposure to our stress exposure paradigm will be needed to explore these possibilities.

Hypothalamus-pituitary-adrenal axis activation is involved in negative feedback control of the immune response as a natural protective mechanism to prevent excessive inflammation (Gill et al., 2009; Koelsch et al., 2016). The uncontrolled and excessive activation of HPA axis in stress disorders is associated with chronic immune system dysregulation (Gill et al., 2009). Indeed, peripheral inflammation has been reported in a number of psychological disorders such as depression (Deslauriers et al., 2018) and PTSD (Gill et al., 2009; Wang et al., 2017). Specifically, IL-1β levels were elevate in combat PTSD males compared to non-traumatized controls (Spivak et al., 1997). In addition, plasma levels of IFN-γ were elevated in PTSD veterans when compared to a healthy control group (Zhou et al., 2014). The low baseline corticosterone levels in our model may underlie a failure of the HPA axis to regulate immune response and therefore explain the elevation in plasma cytokine levels which may contribute toward a primed inflammatory state (**Table 1**).

Exposure to severe traumatic stress is associated with reduced hippocampus volume (Kim et al., 2015; Ahmed-Leitao et al., 2016; Fragkaki et al., 2016; McEwen et al., 2016). A recent large-scale multisite neuroimaging study involving 1868 subjects found smaller hippocampi volumes in subjects with PTSD compared to trauma-exposed controls (Logue et al., 2018). In addition, a meta analysis of magnetic resonance imaging studies of adults with PTSD found hippocampal volume reductions in subjects with PTSD (*n* = 846) compared to traumatized controls (*n* = 624) with the left-hippocampi showing greater reductions (O’Doherty et al., 2015). For the majority of these PTSD subjects, MRI scans were taken more than 10 years since the last trauma (O’Doherty et al., 2015). However, a number of studies have suggested that smaller hippocampal volume is a risk factor for the development of PTSD rather than a trauma consequence (Gilbertson et al., 2010; van Rooij et al., 2015). In animals, chronic restraint stress has been shown to selectively reduce hippocampal volume in rats (when compared to the pre-stress volume) (Gilbertson et al., 2010). A recent study showed that chronic unpredictable restraint stress or diminished adult neurogenesis can contribute to reduced volumes of both dorsal and ventral hippocampus in rats (Schoenfeld et al., 2017). Our results showed a trend toward reduction in the dorsal hippocampus, and a statistically significant reduction in the CA1 of the right dorsal hippocampus 6 months after exposure to RUS. The pyramidal cells of the CA1 subfield of the dorsal hippocampus is involved in processing and retrieval of visuospatial cues or memories (Ji and Maren, 2008), and it relays information received from other hippocampal subfields to neocortical areas for higher cognitive processing. The impact of stress on different subfield regions within the hippocampus remains inconclusive. A study on rats found a reduction in the volume of CA1 after 2 weeks of chronic unpredictable mild stress, while a number of studies found a reduction in the CA3 volume after CUS (McEwen et al., 2016; Morey et al., 2016; Schoenfeld et al., 2017). Impairments within the hippocampal neurocircuitry could contribute toward abnormalities in the encoding, retrieval and processing of memory in PTSD pathobiology; specifically with regard to the ability to discriminate between safe and aversive contexts, i.e., fear generalization. Additional experiments in this current model will be required to explore this further using context specific cues that are needed to evaluate fear generalization. In addition, examining the effect of RUS on fear memory recall compared to a control group receiving only fear conditioning is necessary to understand the effect of extinction on recall of contextual and cued fear memories in the current model.

Brain-derived neurotrophic factor and its receptor TrkB are densely expressed in the hippocampus and their signaling is involved in a variety of functions including supporting neuronal survival, growth, and differentiation (Phillips, 2017). The BDNF/TrkB system has been implicated in the pathophysiology of a number of psychiatric disorders, such as schizophrenia (Autry and Monteggia, 2012), depression (Autry and Monteggia, 2012), and PTSD (Grassi-Oliveira et al., 2008; Angelucci et al., 2014; Dretsch et al., 2016). A single nucleotide polymorphism in the BDNF gene, causing a valine to methionine substitution (V66M) is linked to poor working memory, reduced hippocampal volume (Brooks et al., 2014) and alterations in fear extinction in humans (Soliman et al., 2010). In animal studies, V66M was associated with increased anxiety and depression-like behavior (Chen et al., 2007). In a rat model of PTSD, exposure to psychosocial stress was linked long-term epigenetic changes in hippocampal BDNF DNA (Roth et al., 2011). In addition, several types of stressors, including restraint stress, social defeat, and electric foot shocks, result in a decrease in BDNF levels in the hippocampus (Lee and Kim, 2010; Yu and Chen, 2011). Consistent with our data showing abnormal passive stress coping behavior in animals exposed to RUS, BDNF levels were significantly reduced in the hippocampus of mice exposed to stress (**Figure 6D**). Given that we did not observe any impairments in hippocampal-related tasks, such as spatial learning, and recall of reference memory (Supplementary Figure S5), and taken together, the lower BDNF levels and the reduced CA1 volume observed herein, suggest that exposure to RUS interferes with a unique yet unknown function of the hippocampus in regulating aspects of fear memory expression and alterations in stress coping strategies (**Figure 8**). Regions such as the prefrontal cortex, amygdala, and hypothalamus which are associated with these functions, can receive and project their connections to the hippocampus, and therefore future studies investigating this complex neurocircuitry may help elucidate the role played by this critical brain region in PTSD.

Acute stress exposure and fear learning are associated with immediate (mins to hrs.) increase in amygdala activity as detected by imaging studies, and neuroplasticity (increased NMDA receptors and downstream synaptic signaling partners) (Yasmin et al., 2016; Balu et al., 2017). We did not examine the immediate effects (mins to hrs.) of stress on amygdala activity, however, the reduced expression levels of NMDA receptors and related neuroplasticity markers observed at the acute timepoint (**Figure 7**) may suggest the presence of compensatory mechanisms to the initial hyperactivity in the amygdala neurocircuitry. The hypothalamus is rich in neurons that secrete numerous neuropeptides that regulate several autonomic functions implicated in regulating neurocircuitry pathways involved in mood and stress management. We conducted molecular profiling of regulatory neuropeptides within the hypothalamus, and observed a significant increase in Orexin-A levels at 6 months after stress (**Figure 5**). Orexin-A is a key regulator of the sleep/wakefulness cycle (Berridge et al., 2009; Tsujino and Sakurai, 2013). Sleep disturbance and occurrence of traumatic nightmares is a common feature of PTSD in humans. A number of studies have drawn a critical link between Orexin system dysregulation and stress/anxiety disorders (Johnson et al., 2010, 2012). Orexins are also believed to be an emerging target for treatment of PTSD (Krystal et al., 2017), however, further examination of Orexin signaling and circuitry is needed to understand their role in PTSD pathophysiology. This finding suggests the suitability of this model as a platform to explore the role of Orexins in stress disorders.

## Conclusion

Herein, we have demonstrated that our novel stress model generated a number of PTSD-associated behavioral changes that correlated with decreased baseline plasma corticosterone and ACTH levels, and diminished hypothalamus FKBP5 expression. These results implicate a blunted HPA axis function, which is a key neurobiological finding in PTSD human subjects. The model also shows a chronic reduction in hippocampal CA1 volume and BDNF levels in addition to early and short-term changes in amygdaloid synaptic plasticity markers. We anticipate that our model will be a good platform to understand the chronic effects of stress ultimately leading to the development of novel therapies.

## Author Contributions

MA, JO, DD, MM, and FC designed the research. MA, JO, CL, PM, MO, and BM performed the research. MA and CC analyzed the data. MA, JO, and FC wrote the paper.

## Conflict of Interest Statement

The authors declare that the research was conducted in the absence of any commercial or financial relationships that could be construed as a potential conflict of interest.
