# Supplementary material for: Chronic Hippocampal Abnormalities and Blunted HPA Axis in an Animal Model of Repeated Unpredictable Stress
Source: Front Behav Neurosci. 2018 Jul 20;12:150. doi: 10.3389/fnbeh.2018.00150 (PMC6062757; doi:10.3389/fnbeh.2018.00150)
Supplement: Supplementary file 1 [file Data_Sheet_1.DOCX]

**Supplementary Material for**

**Chronic hippocampal abnormalities and blunted HPA axis in an animal model of repeated unpredictable stress**

**Moustafa Algamal^1,2^**, Joseph O. Ojo^1,2^, Carlyn Lungmus^1,3^, Phillip Muza^1^, Constance Cammarata^1^, Margaret J. Owens^1^, Benoit Mouzon^1,3^, David M. Diamond^4^, Michael Mullan^1, 2^ and Fiona Crawford^1,2,3^

^1^Roskamp Institute, Sarasota, Florida;

^2^The Open University, Milton Keynes, United Kingdom

^3^James A. Haley Veterans’ Hospital, Tampa, Florida;

^4^University of South Florida, Tampa, FL

*To whom correspondence should be addressed: malgamal@roskampinstitute.net

## Supplementary Figures


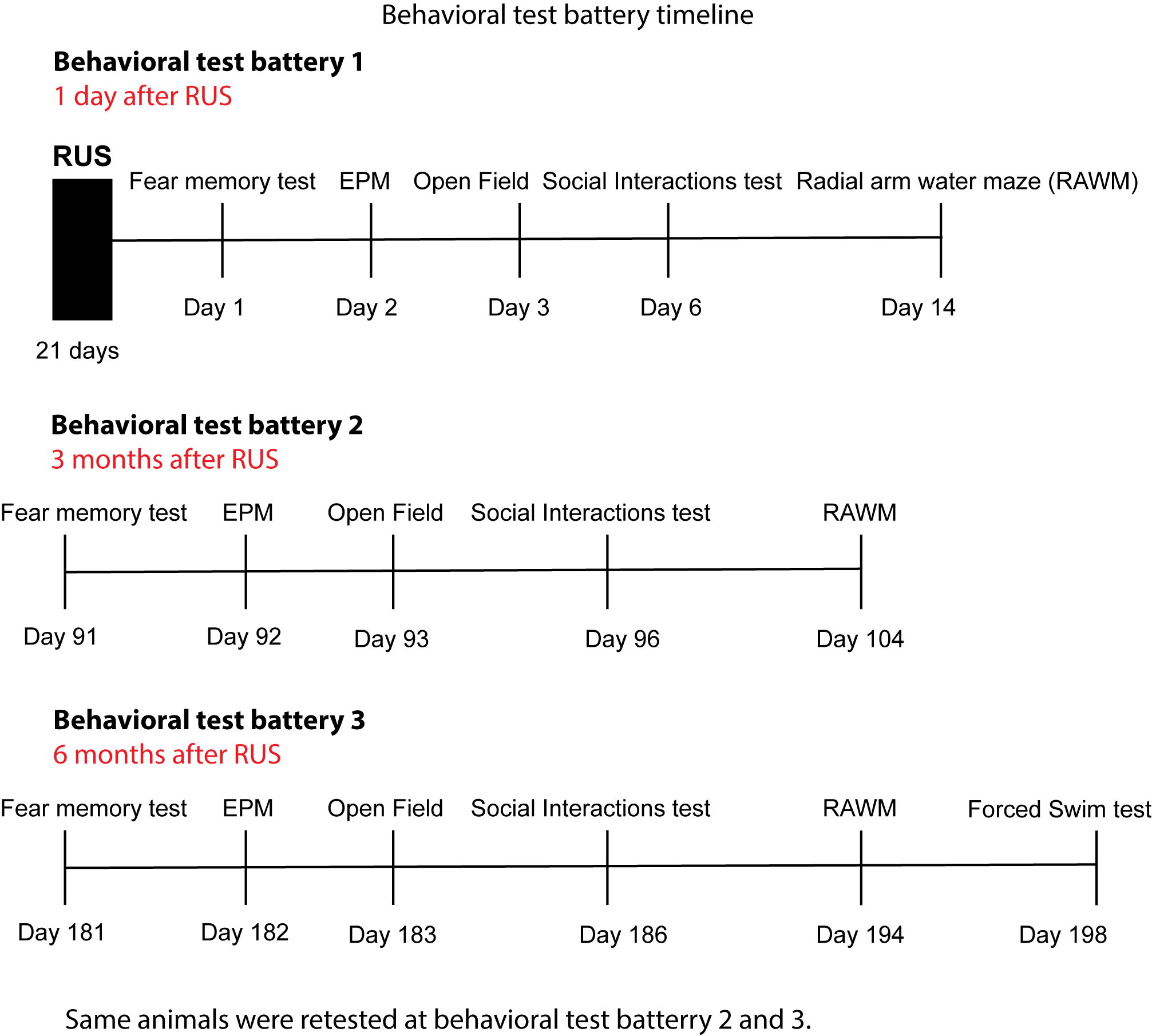


**Figure S1. Timeline of behavioral test batteries.** The same animals tested in behavioral test battery 1 were retested at behavioral test batteries 2 and 3.

**
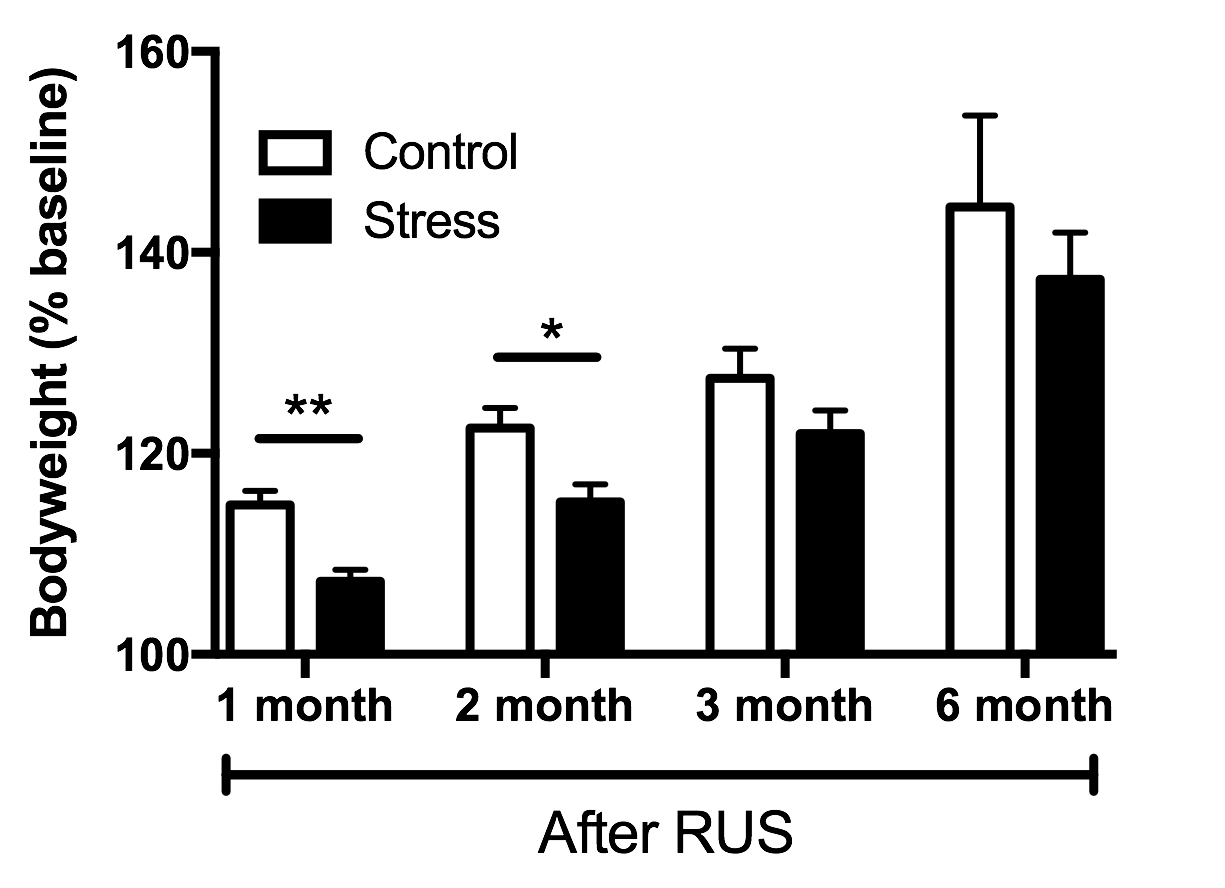
**

**Figure S2 Effect of stress on bodyweight at the chronic timepoints.** Changes in bodyweight persisted till 2 months after RUS. Data were analyzed using a student t-test (n=6-12)..


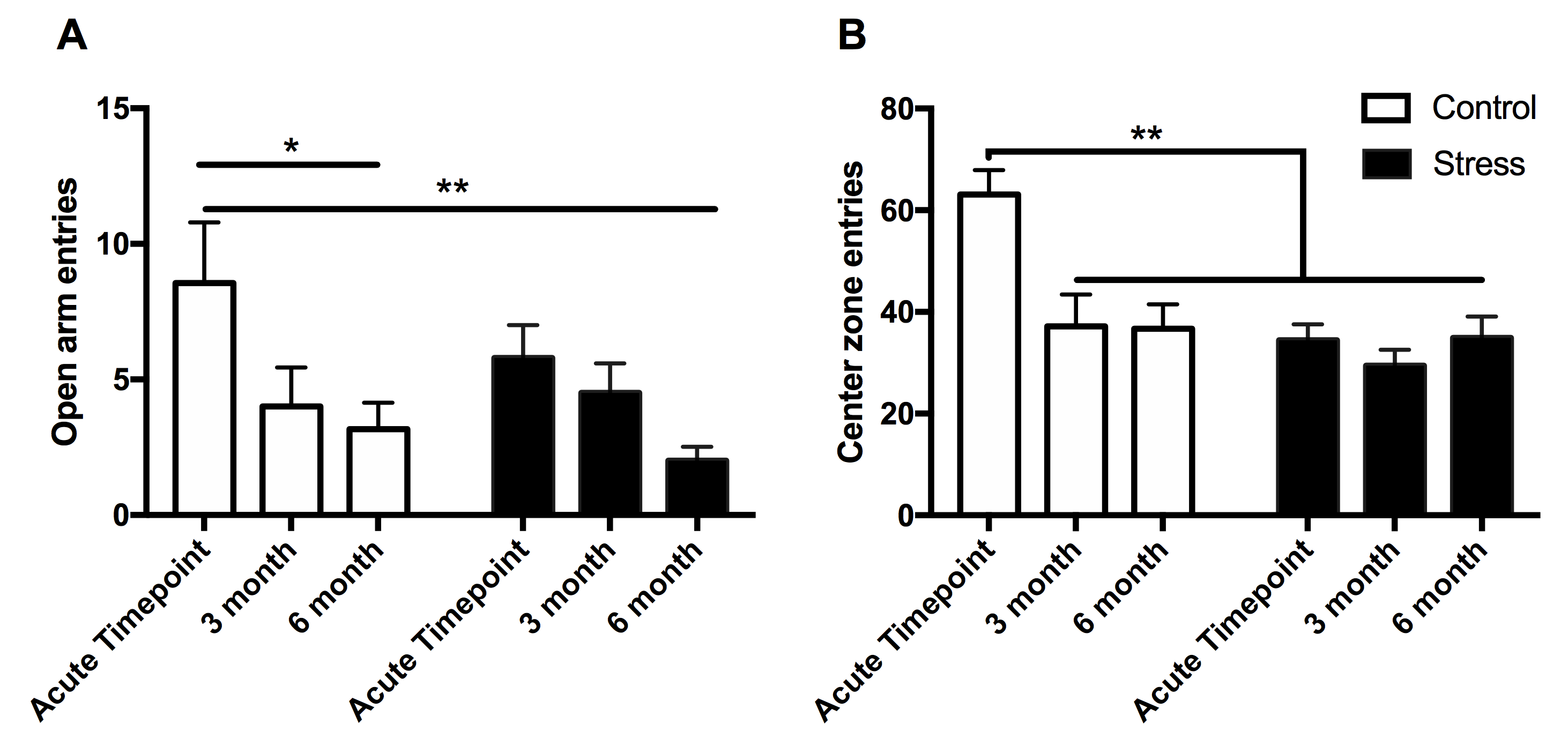


**Figure S3. Effect of repeated testing on mice performance in the EPM and Open Field tests.** (A) Control mice made fewer entries to the open arm of the EPM upon retesting at 6 months after RUS. In the Open field test (**B)**, control mice made fewer entries to the center zone when retested at 3 months and 6 months after RUS. No significant differences were observed between control and stressed animals in both tests at the 3 month and the 6 month timepoints. Data were analyzed using Two-Way ANOVA.


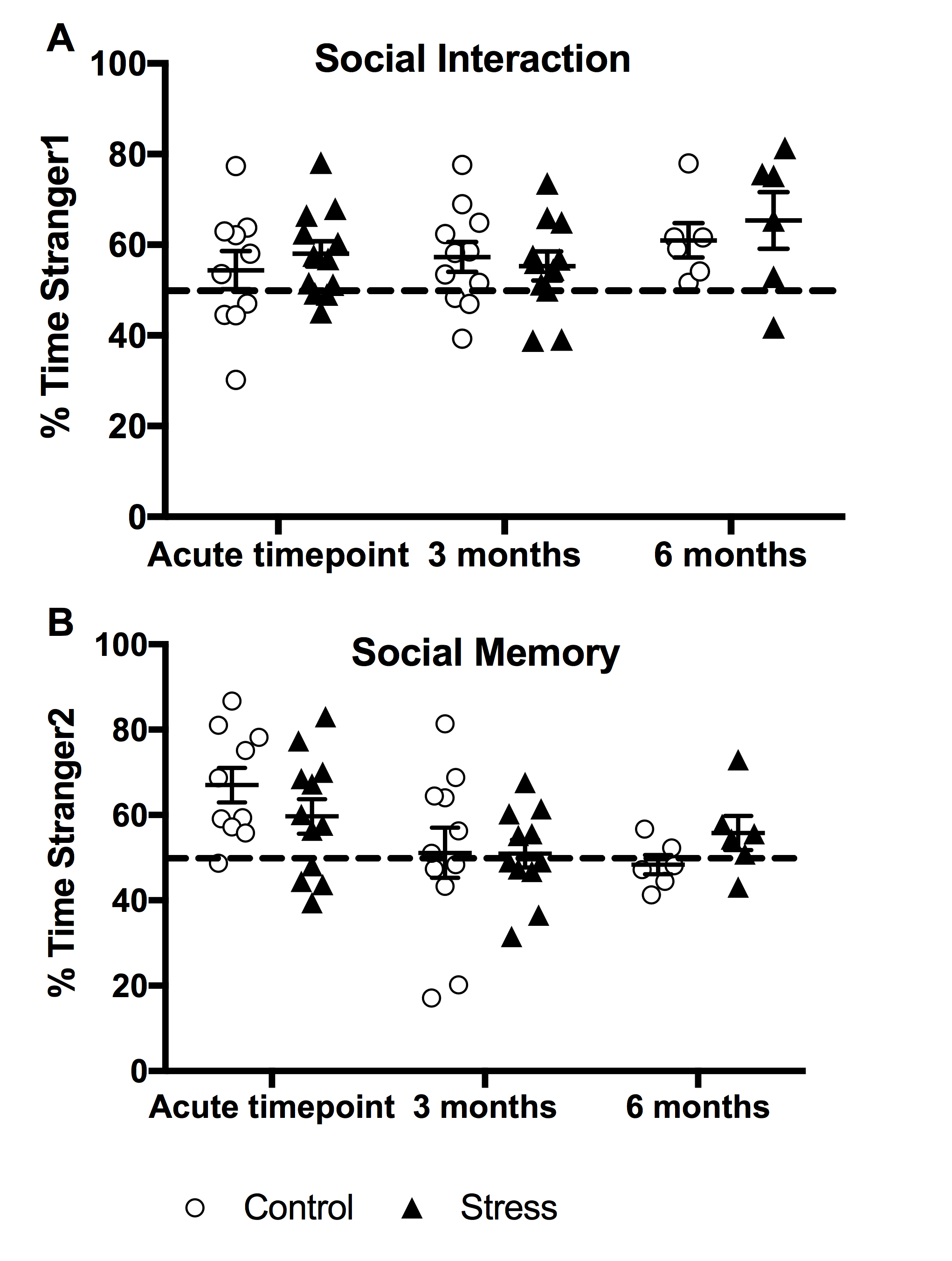


**Figure S4. Effect of stress on social interactions and social memory at the acute timepoint, 3 months and 6 months after RUS.** No differences in social interactions (A) or social memory (B) were observed between RUS animals and controls at the acute timepoint, 3 months or 6 months after RUS. In (A), the ratio between time spent around stranger1 cage to total time spent around stranger1 and the empty cage was calculated for each animal to evaluate social interactions, while in (B), the ratio between time spent around stranger2 cage (new never seen mouse) to the total time around both strangers was calculated to evaluate social memory (social novelty recognition).


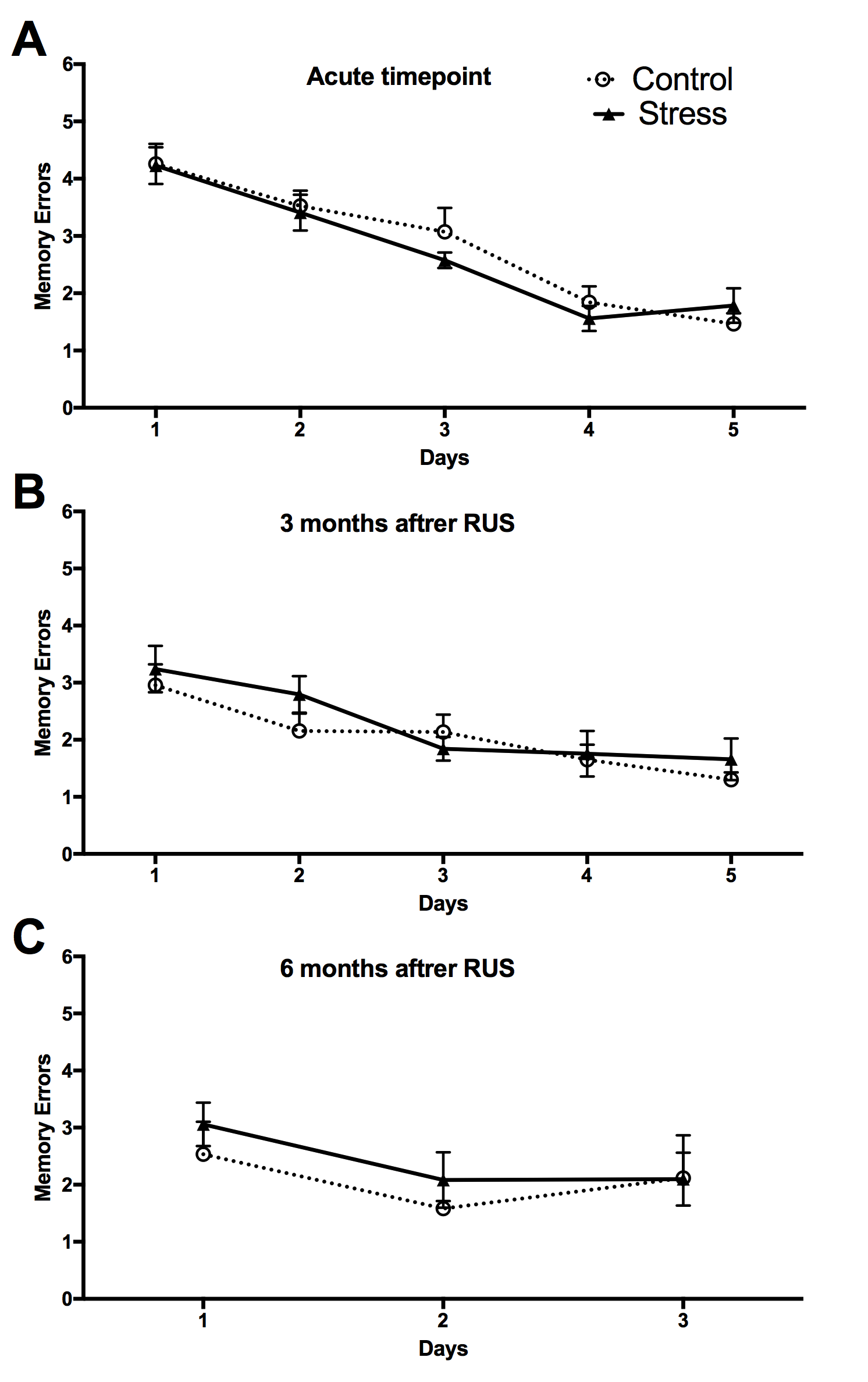


**Figure S5. Effect of stress on spatial learning and memory at the acute timepoint, 3 months and 6 months after last stress**. Panels A, B and C depict the performance of each group during three separate training sessions in a radial arm water maze (RAWM) at the acute timepoint, 3 months and 6 months after last stress. All the animal groups demonstrated a learning curve at all timepoints after trauma. No significant differences between the tested groups were observed at any timepoint. Data were analyzed using repeated measures Two-Way ANOVA. Tukey’s multiple comparisons post-hoc test was performed in all cases.


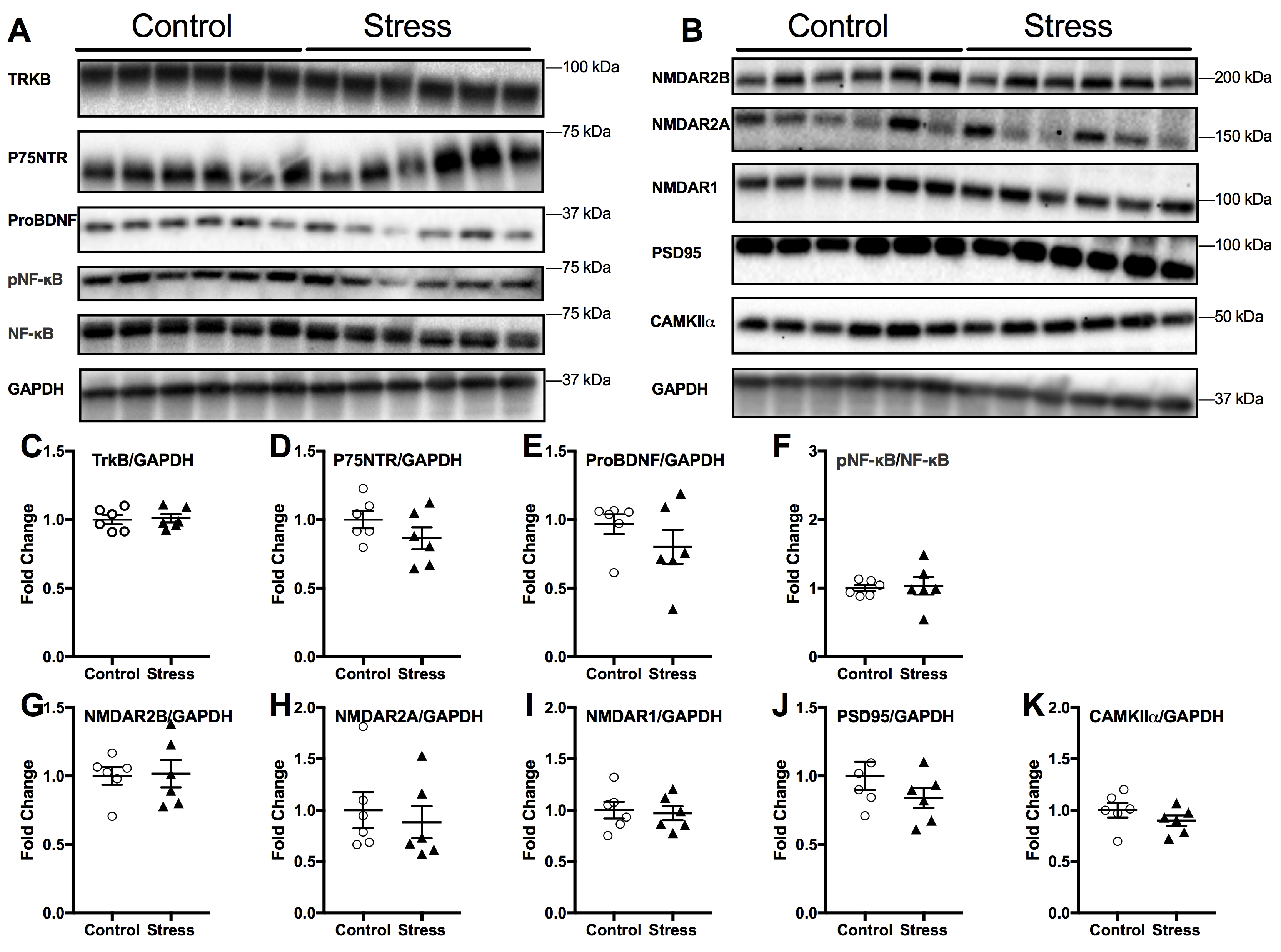


**Figure S6. Effect of stress on synaptic plasticity markers in the hippocampus at 6 months after RUS***.* Representative western blot images of a number of synaptic plasticity from hippocampal lysates markers are shown in panels (A), and (B). Quantification of western blot images of TRKB (C), P75NTR (D), ProBDNF (E), pNF-κB/NF-κB (F), NMDAR2B (G), NMDAR2A (H), NMDAR1 (I), PSD95 (J), and CAMKII (K) levels in the hippocampus at 6 months after RUS. All Data were analyzed using a student t-test (n=6).


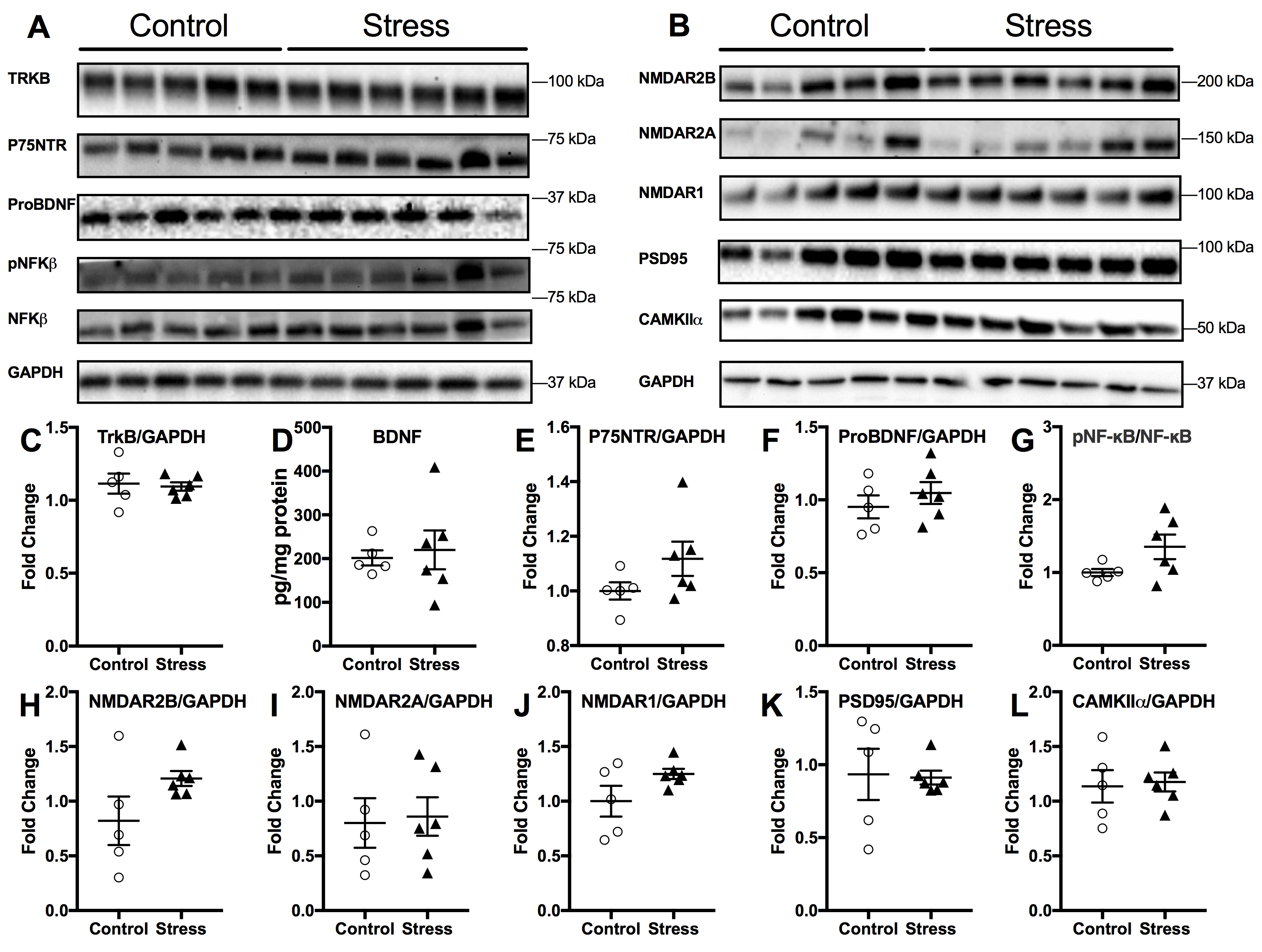


**Figure S7. Effect of stress on synaptic plasticity markers in the amygdala at 6 months after RUS***.* Representative western blot images from amygdala lysates for neurotrophic markers, NMDA receptors and other synaptic plasticity markers are shown in panels (A), and (B). Quantification of western blot images of TRKB (C), P75NTR (F), ProBDNF (F), pNF-κB/NF-κB (G), NMDAR2B (H), NMDAR2A (I), NMDAR1 (J), PSD95 (K), and CAMKII (L) levels in the amygdala at 6 months after RUS. All Data were analyzed using a student t-test (n=5-6).

**Supplementary Table 1. Summary of antibodies (ab) used in western blot experiments**

| Target | 1ry ab dilution | Source | Catalogue # | Host | 2ry ab^$^ dilution |
| --- | --- | --- | --- | --- | --- |
| MR | 1:1000 | Milipore Sigma | MABS496 | Mouse | 1:2000 |
| GR | 1:1000 | Cell Signaling Technology | 12041 | Rabbit | 1:2000 |
| FKBP51 | 1:1000 | abcam | ab2901 | Rabbit | 1:2000 |
| CRH | 1:1000 | abcam | ab184238 | Rabbit | 1:2000 |
| TrkB | 1:2000 | abcam | Ab187041 | Rabbit | 1:2000 |
| P75NTR | 1:1000 | Cell Signaling Technology | 8238 | Rabbit | 1:2000 |
| ProBDNF | 1:500 | Milipore Sigma | MABN110 | Mouse | 1:2000 |
| NMDAR1 | 1:1000 | GeneTex | GTX133097 | Rabbit | 1:2000 |
| NMDAR2A | 1:500 | Thermo Fisher Scientific | PA5-27921 | Rabbit | 1:2000 |
| NMDAR2B | 1:1000 | GeneTex | GTX109713 | Rabbit | 1:2000 |
| PSD95 | 1:2000 | Thermo Fisher Scientific | MA1-046 | Mouse | 1:2000 |
| CAMKIIα | 1:1000 | GeneTex | GTX27939 | Rabbit | 1:2000 |
| NF-κB | 1:1000 | Cell Signaling Technology | 8242 | Rabbit | 1:2000 |
| pNF-κB | 1:500 | Cell Signaling Technology | 3031 | Rabbit | 1:2000 |
| GAPDH | 1:1000 | Cell Signaling Technology | 5174S | Rabbit | 1:2000 |

^$^Anti-mouse and anti-rabbit secondary antibodies were ordered from Cell Signaling Technology
